# Overcoming Lung Challenges in TA-NRP Assisted Heart Recovery in Donation After the Circulatory Determination of Death

**DOI:** 10.3389/ti.2024.13526

**Published:** 2024-11-19

**Authors:** Mario Royo-Villanova, José Moya Sánchez, Alejandro Ortín Freire, Jose H. De Gea García, Sergio Rebollo Acebes, Alba Moreno Flores, Juan Blanco Morillo, Sergio Cánovas, Beatriz Domínguez-Gil

**Affiliations:** ^1^ Intensive Care Unit, Virgen de la Arrixaca University Hospital, Murcia, Spain; ^2^ Cardiovascular Surgical Unit, Virgen de la Arrixaca University Hospital, Murcia, Spain; ^3^ National Transplant Organization, Madrid, Spain

**Keywords:** NRP, thoracoabdominal normothermic regional perfusion, heart donors, lung, ECMO

## Abstract

Thoraco-abdominal normothermic regional perfusion (TA-NRP), utilizing Extra Corporeal Membrane Oxygenation (ECMO) devices, has emerged as an effective strategy for heart recovery in donors declared dead by circulatory criteria (DCDD). After death declaration, TA-NRP restores heart activity by reperfusing the arrested heart with oxygenated blood at normothermia. Mechanical ventilation resumption in the donor enables weaning from ECMO and restores systemic circulation and oxygenation using the donor’s heart and lungs. However, if pre-existing conditions prevent the donor’s lungs from oxygenating blood post-cardiac activity restoration, weaning from veno-arterial ECMO may lead to systemic hypoxia, jeopardizing the restored cardiac function. Anticipating this scenario may guide planning a split ECMO circuit to facilitate earlier and more effective recovery of donor heart function post-ECMO weaning. This manuscript describes three cases of DCDD donors with hypoxic respiratory failure undergoing TA-NRP for heart recovery. By establishing a bridge in the arterial portion of the circuit, clamped out after weaning from veno-arterial ECMO, donor heart function was assessed exclusively with veno-venous ECMO support, leading to successful heart transplantation.

## Graphical Abstract

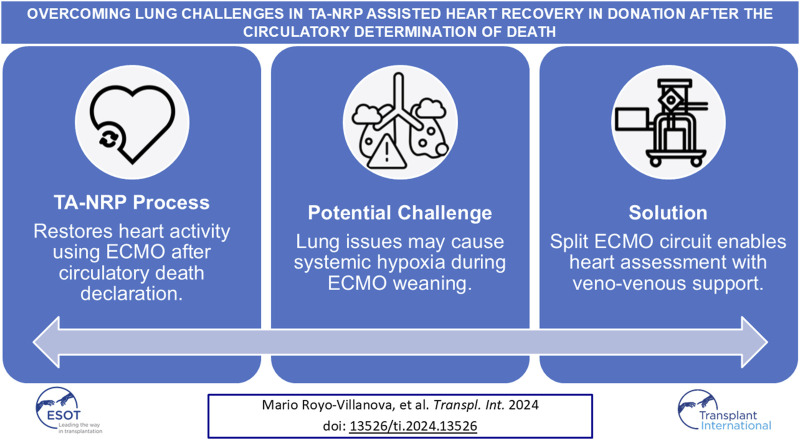

## Introduction

Heart recovery in donation after circulatory determination of death (DCDD) represents a recent advancement that expands the pool of hearts available for transplantation. Outcomes for heart transplants from DCDD donors appear comparable to those from donors declared dead by neurological criteria [1] (DNDD). As the demand for heart transplants continues to grow, innovative strategies like DCDD are critical to increasing the availability of viable donor hearts.

The utilization of thoraco-abdominal normothermic regional perfusion (TA-NRP), initially introduced by the Papworth group [2, 3], has emerged as an advantageous method for *in situ* reperfusion and restoration of activity in the asystolic heart. This technique, based on the use of Extra Corporeal Membrane Oxygenation (ECMO) devices, enables the functional evaluation of the heart after the declaration of death and prior to organ recovery. TA-NRP allows restoring heart activity by reperfusing the arrested heart with oxygenated blood at normothermia. Once heart activity is restored, the reinstatement of mechanical ventilation in the donor facilitates the weaning of ECMO, allowing systemic circulation and oxygenation via the donor’s own cardiac and pulmonary functions.

However, challenges arise when the donor has succumbed to severe and advanced lung disease or severe hypoxemic respiratory failure. In such cases, the donor’s lung function may be insufficient to provide proper systemic oxygenation, thus hindering adequate cardiac contractility. Anticipating and managing these scenarios is crucial for the successful recovery and transplantation of donor hearts.

This manuscript reports a series of three DCDD donors with hypoxic respiratory failure prior to death where heart recovery was successfully performed through TA-NRP using a specific technical approach. We describe the methodologies used, the clinical outcomes, and discuss the implications of these findings for future DCDD heart transplantation protocols.

## Methods

### TA-NRP Protocol

In Spain, the national DCDD heart transplant protocol [4] is based on the use of TA-NRP for the recovery and *in situ* assessment of heart viability. This assessment is performed using transesophageal echocardiography and/or Swan-Ganz catheterization. Once recovered, the DCDD heart is subject to static cold storage before transplantation.

In the standard procedure, after the provision of life support measures, waiting to circulatory arrest, a 5-min “no-touch” period is observed, and the patient is declared deceased. Subsequently, a rapid sternotomy is performed with clamping and sectioning of the supra-aortic trunks to avoid restarting brain flow [5], followed by the initiation of TA-NRP.

Upon restoration of the donor heart’s activity, it is crucial to wait for the restoration of optimal cardiac function capable of producing adequate systemic output to perfuse both the coronary circulation and the donor’s other organs. Typically, with the commencement of TA-NRP, heart function experiences prompt recovery, enabling the gradual weaning of TA-NRP. Ongoing assessment of donor heart function is performed using a transesophageal echocardiogram, which is particularly valuable in evaluating cardiac performance in the now heart-beating donor.

To facilitate a gradual transition from normothermic regional perfusion, reintubation of the recipient and gradual oxygenation of their blood using the donor’s native lungs allow for the gradual weaning of ECMO support.

### TA-NRP in Donors With Hypoxic Respiratory Failure

**FIGURE 1:**
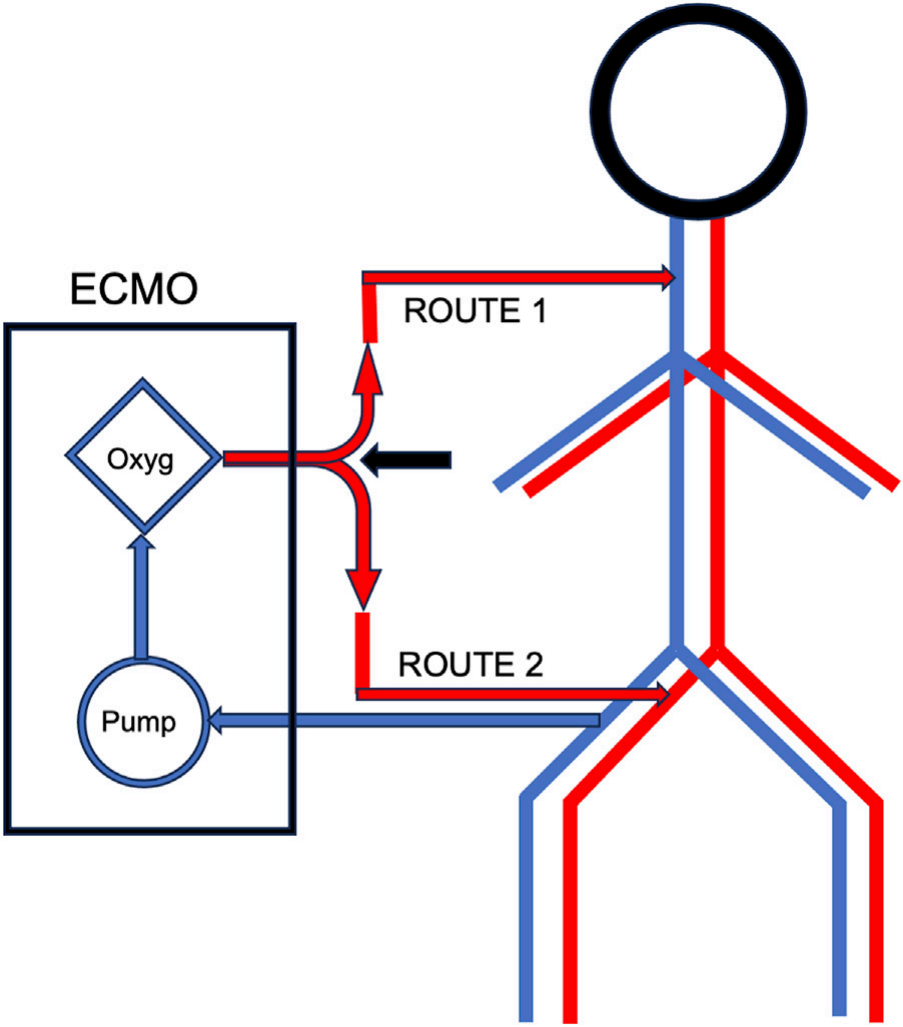
ECMO Y-shape bypass.

In scenarios where donors have severe hypoxic respiratory failure, a Y-shaped bypass integrated into the ECMO circuit can be used to allow quick diversion of the loop and immediate re-conversion from veno-arterial TA-NRP to veno-venous TA-NRP (Figure 1). This configuration is achieved using a simple diverting clamp, which permits *in situ* oxygenation of the donor’s organs when the donor’s lungs are insufficient to perform this function independently.

We report a series of three DCDD donors with hypoxic respiratory failure prior to death and cardiac procurement. Written informed consent for their recruitment and publication of data was authorized by close family members and the study was approved by the Ethics Committee for Medical Research at Virgen de la Arrixaca University Hospital (CEIM).

## Results

Two of the donors were already on VV ECMO support and had a femoral arterial cannula placed for NRP prior to the withdrawal of life-sustaining therapies (WLST). The third donor experienced rapid respiratory deterioration immediately before WLST and was placed on VA ECMO as a bridge to convert to VV ECMO.

The first two cases had femoral and jugular veno-venous ECMO, requiring only the insertion of an additional femoral arterial cannula. In the third case, separate arterial and venous cannulas were inserted into the femoral vessels, along with an additional cannula in the right jugular vein. Thus, all donors had three cannulas before WLST (Spanish legislation allows pre-mortem preservation maneuvers with appropriate family consent). The ECMO circuit was initially connected to the femoral vessels (veno-arterial) to restart systemic circulation once the patients were declared dead.

In all cases, cardiac activity resumed within 2 minutes after initiating TA-NRP. Following adequate initial cardiac contractility, attempts to withdraw VA TA-NRP resulted in a significant and immediate decrease in contractility and cardiac output, accompanied by severe hypoxia. Through the Y-bypass of the ECMO, the NRP circuit flow was redirected to veno-venous by extracting blood via the femoral venous route and re-infusing oxygenated blood via the jugular vein. Under these conditions, cardiac contractility and output were maintained optimally until organ validation and subsequent perfusion with cold cardioplegia solution.

All three hearts were deemed suitable for transplantation, and all three were successfully implanted in the recipients (Table 1).

**TABLE 1:** Outlines the clinical characteristics of the cases.

Donor								Recipient						
	Age	ICU days	Diagnosis	ECMO (days)	Weight	Height	CIT	VA ECMO time	VV ECMO time	LVEF at retrieval	PIcuD	PIS	PDS	DAFH
Patient 1	51	12	ILS	11	100	180	96	16	21	65	13	Yes	No	Yes
Patient 2	33	39	Polytrauma (thoracic trauma)	36	92	182	108	23	33	65	9	Yes	No	Yes
Patient 3	42	12	Polytrauma (VAP)	0	75	170	73	12	18	60	16	Yes	No	Yes

ILS: interstitial lung disease, VAP: Ventilator-Associated Pneumonia, Age (years), Weight (Kilograms), Height (centimeters), CIT: Cold ischemic time (minutes), VA ECMO, time (minutes), VV ECMO, time (minutes), LVEF: left ventricular ejection fraction, PIcuD: postoperative ICU, days, PIS: postoperative inotropic support, PDS: postoperative device support, DAFH: discharge alive from the hospital.

## Discussion

The successful recovery of hearts from DCDD donors with severe hypoxic respiratory failure demonstrates the viability of using TA-NRP in combination with a modified ECMO circuit. If, due to any pre-existing condition, the donor’s lungs are unable to oxygenate the blood after restoring cardiac activity, weaning from VA-ECMO may be impossible due to systemic hypoxia, compromising the newly restored cardiac activity.

When there is suspicion that mechanical ventilation of the lungs may be insufficient to oxygenate the blood of the DCDD donor, such as in cases previously supported with VV-ECMO, it is critical to preserve the VV-ECMO circuit. This ensures adequate oxygenation during the weaning of VA-ECMO by quickly reconfiguring it to VV-ECMO with a double bypass. Anticipating the insertion of a second jugular venous cannula in potential donors not already on VV-ECMO, but exhibiting severe hypoxia prior to the withdrawal of life support measures, can facilitate the conversion of the femoro-femoral VA circuit to a femoro-jugular VV circuit if necessary. This approach can be highly beneficial for heart resuscitation and successful transplantation.

The findings from these cases suggest that preemptive strategies and modifications to the ECMO circuit can significantly enhance the outcomes of DCDD heart recovery, even in donors with compromised pulmonary function. Future protocols for DCDD heart transplantation should incorporate these techniques to maximize the pool of eligible donors and improve transplantation success rates.

The use of TA-NRP combined with strategic modifications to the ECMO circuit can effectively overcome the challenges posed by donors with severe hypoxic respiratory failure. By ensuring adequate oxygenation and maintaining cardiac function during the weaning process, it is possible to achieve successful heart transplantation outcomes. These findings underscore the importance of tailored approaches in DCDD heart recovery and highlight the potential for expanding the donor pool through innovative techniques.

## Data Availability

The raw data supporting the conclusions of this article will be made available by the authors, without undue reservation.
